# An external telemetry system for recording resting heart rate variability and heart rate in free-ranging large wild mammals

**DOI:** 10.1371/journal.pone.0252013

**Published:** 2021-06-04

**Authors:** Sean D. Twiss, Naomi Brannan, Courtney R. Shuert, Amanda M. Bishop, Patrick. P. Pomeroy, Simon Moss

**Affiliations:** 1 Department of Biosciences, Durham University, Durham, United Kingdom; 2 Sea Mammal Research Unit, Scottish Oceans Institute, University of St. Andrews, St. Andrews, United Kingdom; Animal Health Centre, CANADA

## Abstract

Measures of heart rate variability (and heart rate more generally) are providing powerful insights into the physiological drivers of behaviour. Resting heart rate variability (HRV) can be used as an indicator of individual differences in temperament and reactivity to physical and psychological stress. There is increasing interest in deriving such measures from free ranging wild animals, where individuals are exposed to the natural and anthropogenic stressors of life. We describe a robust, externally mounted heart rate monitor for use in wild mammals, deployed here on wild breeding adult female grey seals (*Halichoerus grypus)*, that delivers millisecond precise measures of inter beat intervals (IBIs), allowing computation of resting HRV parameters. Based on Firstbeat™ heart rate belts, our system allows for remote, continuous recording of IBI data from over 30 individuals simultaneously at ranges of up to 200m. We assessed the accuracy of the IBI data provided by the Firstbeat™ system using concurrent IBI data derived from in-field electrocardiogram (ECG) recordings. Bland-Altmann analyses demonstrated high correspondence between the two sets of IBI data, with a mean difference of 0.87±0.16ms. We used generalized additive mixed-effects models to examine the impact of the default Firstbeat™ software artefact correction procedure upon the generation of anomalous data (flats and stairs). Artefact correction and individual activity were major causes of flats and stairs. We used simulations and models to assess the impact of these errors on estimates of resting HRV and to inform criteria for subsampling relatively error free IBI traces. These analyses allowed us to establish stringent filtering procedures to remove traces with excessive numbers of artefacts, including flats and stairs. Even with strict criteria for removing potentially erroneous data, the abundance of data yielded by the Firstbeat™ system provides the potential to extract robust estimates of resting HRV. We discuss the advantages and limitations of our system for applications beyond the study system described here.

## Introduction

Resting heart rate variability (HRV; variation in the intervals between heartbeats) has proven to be an informative physiological measure, integrating a wide range of processes linked to sympathovagal balance into a metric that is indicative of an individual’s temperament or coping style [1–3] and reactivity to stress, whether psychological or physical [3–6]. Acquisition of reliable measures of resting HRV can be more challenging than estimates of heart rate due to the requirement for millisecond precision of inter-beat interval (IBI) data through accurate detection of RR peaks [3, 5]. Although electrocardiogram (ECG) devices provide the ‘gold standard’ for recording HRV metrics due to their ability to record the entire QRS complex, they are rarely practicable in studies of freely moving animals [7, 8]. Even non-invasive portable ECG devices, such as Holter monitors [9, 10], are typically only capable of short-term recordings making them more suitable for captive or laboratory animals and livestock. This places constraints upon the ability of researchers to incorporate the potentially useful HRV metrics in studies of free-ranging wild animals. Therefore, there is a need for affordable robust devices that can provide reliable IBI data at the required millisecond precision on free-ranging wild animals. Ideally, such devices would allow for long-term and remote recording of data across multiple individuals to minimise disturbance from the number of interventions required, allowing the study animals to behave normally in their natural environment. We describe and assess a system that has the potential to fulfil this role even in a challenging situation.

Increasingly, heart rate monitors are being integrated into wild studies, where animals are exposed to the natural challenges and stressors of life [11–15]. The telemetry devices used often differ depending upon research context and study species, with differences in morphology or life history often dictating the best strategy to employ [14–16]. For example, some studies utilize implanted electrodes to record heart rate or HRV [17, 18]. These systems not only require recovery of devices for data-download, but in non-sterile conditions it is often desirable to avoid surgical implantation of devices, thus, externally mounted heart rate monitors can provide a viable solution.

External telemetry devices, such as the Polar® RS800CX with H2/H3 sensors (Polar Electro Oy, Kempele, Finland), can provide the required millisecond precision by using peak voltage to register the time-points of successive R-peaks. IBI data are transmitted from the sensor mounted on the study individual (with appropriate electrodes) to the remote RS800CX receiver. Such devices can permit prolonged monitoring of IBIs in freely moving subjects and are typically more affordable than ECG devices, permitting larger sample sizes. However, with a limited transmission range (c. 20-50m), the majority of studies utilising this system have deployed devices on relatively constrained individuals; either in laboratories or captivity, or on domesticated species in controlled conditions [4, 5, 12, 13, 19]. Understandably, relatively few studies have attempted to deploy such heart rate monitors to measure HRV in a wild context [6, 8, 20].

A major consideration with externally mounted heart rate monitors is that they can be prone to errors (artefacts) in IBI data [3, 5, S1 Fig in S1 File]. Artefacts in IBI data can lead to significant biases in estimates of HRV and dealing with artefacts is an important stage in ensuring reliable metrics of HRV are generated [3, 5, 21]. Sources of artefacts in IBI data can be intrinsic (e.g. arrhythmias, noise from muscle action potentials), or extrinsic (e.g. poor electrode-skin conductance, equipment malfunction), causing beats to be either missed or spuriously generated, leading to erroneously long or short IBI values [3, 5, 12]. Artefacts generated by spuriously long or short IBIs create extreme ‘peaks’ (or troughs) in a trace of IBIs, and can be detected and corrected using a range of software (e.g. “Kubios” [22], “ARTiiFACT” [23], “RHRV” [24]) although thresholds must be considered carefully to avoid removal of normal biological variation in IBIs such as sinus arrythmia. Correction is typically done by means of deleting spurious beat(s) or replacing missing beat(s) through interpolation. However, there are two forms of artefacts that cannot be corrected by these software packages [5, 8, 12]: The first presents as invariable sequences of IBIs, such that graphical representations of IBIs over time form a flat, horizontal line. For simplicity we refer to this form of artefact as ‘flats’. The second are sequences of monotonically increasing or decreasing IBIs, which appear as straight, positively or negatively, sloped lines on graphs of IBIs against time. We refer to these as ‘stairs’. Further details of the form of flats, stairs and other artefacts are provided in S1 Fig in S1 File. Few studies have explicitly considered the impacts of flats or stairs on the resulting estimates of resting HRV. One study [8] stated that any trace where >5% of the trace was classified as any type of error should not be used for estimating HRV. Such strict criteria for removing potentially erroneous data can be utilised in captive situations where a researcher is likely to have the option to re-measure a restrained animal. However, in field studies such options for re-deployment are unlikely to be available, and ethical considerations drive a need to maximise the utility of any collected data. In addition, determination of resting HRV, the key baseline metric of individual coping style, requires subjects to be in a resting state primarily [3]. Field studies of free ranging animals therefore also require some means of determining the activity of their study individuals and temporally linking this information to IBI data.

Here we describe a robust, externally mounted system based on Firstbeat™ heart rate belts (https://international-shop.firstbeat.com/product/team-pack/) capable of long-term remote recording of IBIs with millisecond precision from multiple free-ranging individuals simultaneously. We deployed this system on wild adult female grey seals (*Halichoerus grypus*) during their annual breeding season. As deep divers, phocid seals, such as the grey seal, are renowned for their remarkable capacity to alter their heart rate (documented heart rate in free-living grey seals ranges from 4 to 120 bpm) [25, 26]. In addition, the terrestrial breeding sites occupied by phocid seals present physically challenging conditions for any externally mounted heart rate monitor, with aggregations of seals interacting, often aggressively, and moving around on a variety of substrates including rock, mud, sand and in water [27]. Our aims in this study are to (i) assess the ability of this system to generate accurate IBI data in a field context, (ii) assess the occurrence and potential causes of artefacts (in particular flats and stairs) within traces generated by this system, and (iii) determine the potential impact of these errors on estimates of resting HRV derived from the IBI data.

## Methods

### Description of the study system, heart rate monitor design and deployments

#### Study animals and study site

Data were collected at the Isle of May (56.1° N, 2.55° W) grey seal breeding colony in 2015, 2016 and 2017 (27/10-24/11/2015; 25/10-27/11/2016; 23/10-11/12/2017). Adult female grey seals typically begin to arrive on this colony in mid-October, with peak density of seals ashore in mid-November [28]. Individual females will spend 18–20 days ashore, during which they each bear and nurse one pup, enter oestrus towards the end of lactation (approximately 16 days after giving birth [29]) and mate. Grey seals are capital breeders, fasting whilst on the colony and relying on energy reserves gathered prior to the breeding season, stored primarily as blubber [30]. The Isle of May has a rugged topography with aggregations of breeding seals scattered across the island but primarily occupying pockets of flatter terrain with access to water (either tidal inlets or pools of water) [27, 28, 31].

#### Capture/handling of seals

Breeding females and their pups are routinely captured as part of long-term reproductive studies by the Sea Mammal Research Unit, SMRU [28, 30, 32]. Individual females were identified using pre-existing brands, flipper tags and/or pelage patterns [30, 33, 34]. Details of the capture procedure are provided elsewhere [30, 35, 36]. In brief, target females of known identity are chemically immobilized using a mass-specific intramuscular injection of zolazepam-tiletamine (‘Zoletil’, Virbac, U.K.). Immobilization was maintained for 30–40 min allowing measurement of maternal mass, morphometrics, collection of physiological samples (e.g. blood) and attachment of telemetry devices. Mothers are captured and sampled twice during their lactation period, once early in lactation and again towards the end of lactation, before the female enters oestrus [29]. Telemetry devices were recovered during the late lactation captures. These double captures permit calculation of maternal mass changes across the main period of lactation [30], and, assuming a linear rate of mass loss, can be used to estimate maternal mass on any day during lactation.

All animal procedures were performed under the UK Home Office project licence #60/4009 and conformed to the UK Animals (Scientific Procedures) Act, 1986. All research received prior ethical approval from the Durham University’s Animal Welfare Ethical Review Board and from the University of St Andrews Animal Welfare and Ethics Committee and the School of Biology’s Ethics Committee. NatureScot (formerly Scottish Natural Heritage) approved field site access to the Isle of May national nature reserve (permit numbers by year; 2015 = MON/RP/175, 2016 = MON/RP/177, 2017 = MON/RP/178).

#### Heart rate monitor design and attachment

Firstbeat™ heart rate belts (Jyväskylä, Finland) are designed for use on humans and use peak voltage to register successive R-peaks in the QRS complex with millisecond precision (sampling rate of 1000 Hz). The devices compute the time between successive R peaks to generate a sequence of R-R intervals or inter-beat intervals (IBIs). The transmitter portion of the belts utilises the ‘BlueRobin’ transmission protocol (868/915 MHz) to transmit IBI data (in milliseconds) to a remote receiver (Firstbeat™ Team Receiver) located at ranges of up to 200 m (line of sight) from the transmitters. The receiver is connected to a PC which logs the IBI data. The Team Receiver is capable of logging IBI data from multiple transmitters, deployed on different individuals, simultaneously. We used the Team Receiver 30, allowing up to 30 simultaneous recordings.

**Fig 1:**
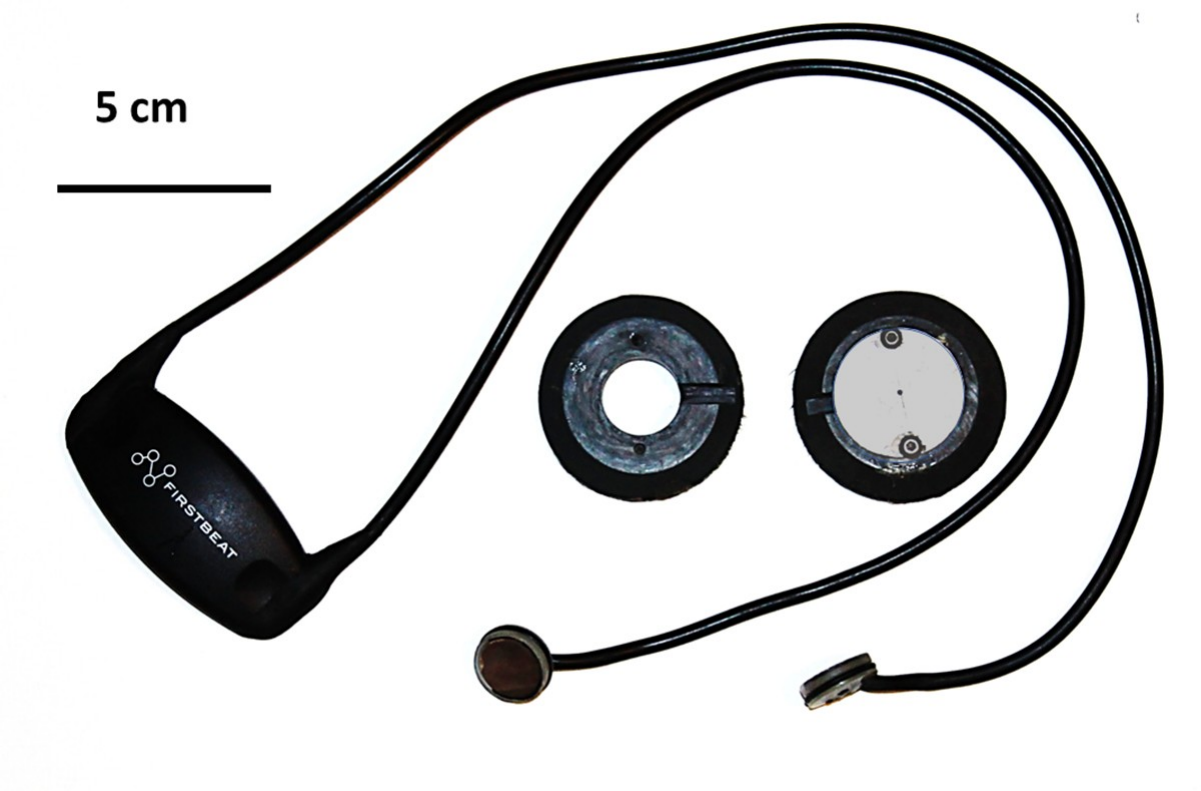
Firstbeat^TM^ transmitter and modified electrodes. Image also shows the two attachment donuts, the right hand one with cover plate in place.

To modify Firstbeat™ heart rate belts to suit our study species (Fig 1), we removed the two short rubberized electrode straps (12 cm x 2.5 cm) extending from the central transmitter unit and replaced them with 50cm protected cables screwed to the strap contact through a small solder tab. Each distal end of the two cables was passed through a hole in a 50 ml adhesive cartridge piston (TAH industries Inc., Robbinsville, NJ) with EDPM O-ring. Cables passed through the piston face at a shallow angle and were silver soldered to a silver disc (16 mm diameter, 0.3 mm thickness, fully annealed sterling silver disks). The disc was then pressed flush with the open side of the piston. The connections made on the Firstbeat™ module and the recess in the piston were then back filled with polyurethane composite after priming. The silver discs and pistons were then treated by immersion in 11% sodium hypochlorite solution to create a silver chloride electrode (Fig 1).

We also fashioned PVC donuts (52 mm outer diameter) with a bevelled outer edge (Fig 1) and a central hole (19 mm diameter) to accept the electrode and plastic cup with its O-ring. The donut had a lateral channel (4 mm wide, 4 mm deep) extending from the inner hole to the edge of the bevelling to allow the electrode cable to sit flush with the top surface of the donut. Two holes were drilled and tapped into the outer flat face of the donut to receive machine screws for attachment of an aluminium cover plate (Fig 1). Firstbeat™ heart rate monitors require CR2032 lithium batteries, and we used new (and tested) batteries prior to each deployment (Maxell CR2032) which were adequate to power the monitors for our maximum deployment duration (13 days). The battery compartment was sealed using waterproof tape. The total mass of the heart rate monitor, donuts and cover plates was 0.11 kg. The minimum re-capture mass for a female grey seal in our study was 100.2 kg, therefore, the attached devices were less than 0.1% of seals body mass.

#### Attachment of the heart rate monitors

**Fig 2:**
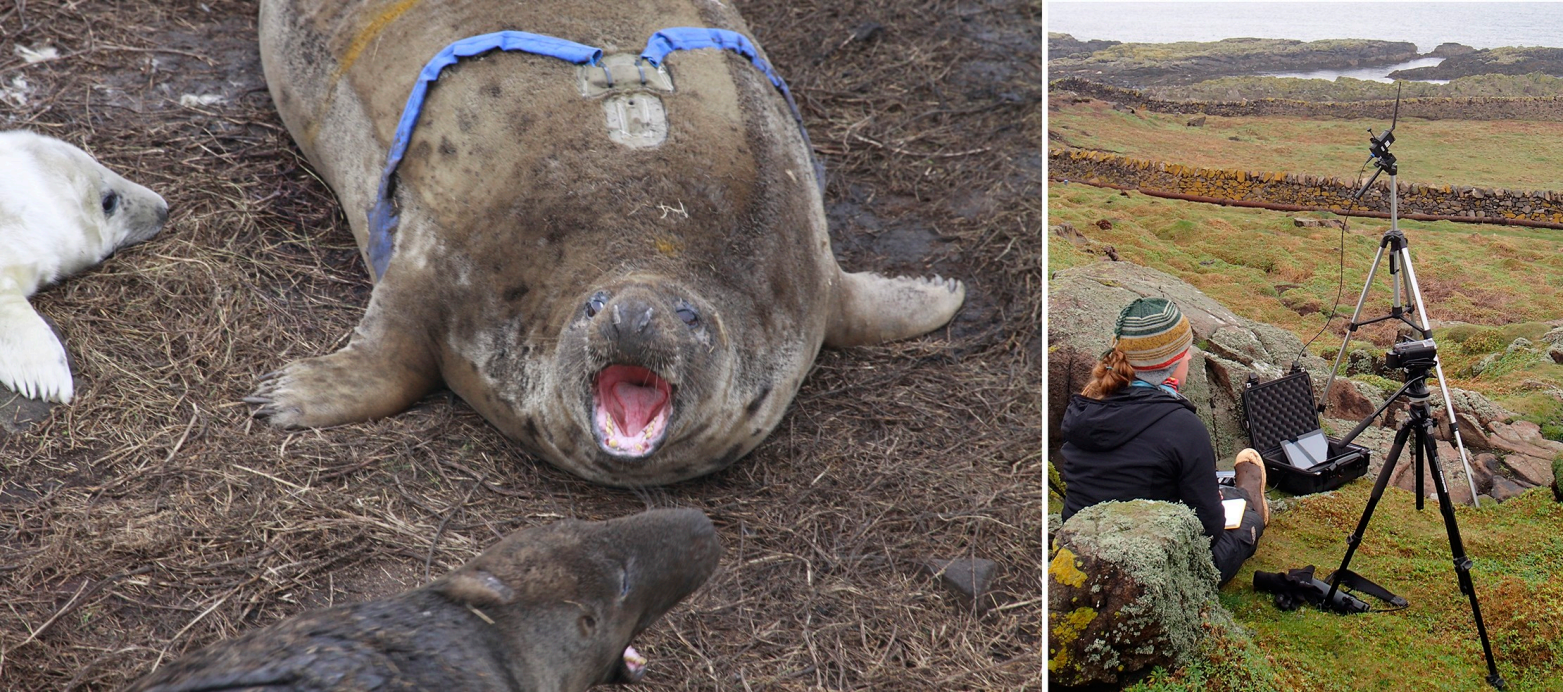
The mounting of the Firstbeat^TM^ transmitter and modified electrodes on an adult female grey seal. Devices and cables were protected by nylon (blue and green in Fig 2A). Anterior of the centrally mounted heart rate transmitter is the accelerometer mounting (a). Data were transmitted to a receiving station located within line-of-sight at distances of up to 200 m from instrumented seals (b).

Seal pelage was cleaned with water and dried prior to attachment. All attachments were made using the adhesive Loctite 422 (Henkel Ltd., Hemel Hemstead, UK) to the upper layer of pelage. For attachment of the transmitter, we designed a bespoke ballistic nylon pocket that was mounted dorsally between the scapulae (Fig 2A), with the opening facing posteriorly. The transmitter was placed within the pocket and secured by means of two cable ties (5 mm width) contained within sewn channels running anterior-posterior in the base of the pocket.

The electrode cables were inserted into ballistic nylon sleeves that extended from the transmitter to within 20 mm of the electrode. We used these nylon sleeves as cable protection, and they permitted attachment of the cables to the fur. Sleeves were glued at approximately 5 cm intervals to the fur to ensure firm attachment while allowing for flexion and torsion exerted by the seal during normal behaviours. The electrodes were then positioned directly posterior of the fore flippers. A circle of fur c.18 mm was clipped to ca. 1 mm hair length using Lindstrom™ Angle Type Wire Cutter (108 mm overall length, 0.35–1.0 mm cutting capacity). The donuts were then glued to the surrounding full-length hair (typically ca.10 mm) so that the inner hole of the donut encompassed the clipped fur, and the cable channel was angled approximately 45^o^ posterior and dorsally. We added electrode gel (Ten20: Weaver and Company, Aurora, Colorado, USA) to the inner hole, and to the base of the electrode to improve conductance. The electrodes were then inserted into the inner hole of the donut and plastic spacers (10 x 10 x 3 mm) were inserted behind the electrodes to ensure proximity of electrode to the skin. Finally, an aluminium cover plate was placed over the donut via two machine screws.

#### In-field deployments

In total, 52 different individuals were instrumented across the three field campaigns. Our aim was to redeploy devices on the same individuals in multiple seasons where possible [37], therefore, across these three seasons we performed 89 separate deployments. The length of deployment ranged from 6 to 13 days, with a mean deployment of 9 days. For in-field recordings of IBIs, the Firstbeat™ receiver was attached to a tripod with antennae height approximately 1.8 m above ground level and positioned 50–200 m away from instrumented seals (Fig 2B). Due to the broken terrain of the Isle of May and patchy distribution of breeding seals not all seals could be recorded simultaneously by line-of-sight. Daily recordings typically involved collection of data on 2–6 seals for 2–4 hours, before relocating the receiver to monitor other individuals. We endeavoured to balance data collection on all instrumented seals across the time of day (daylight hours only) and the deployment period. We conducted direct behavioural observations concurrent with logging IBI data [38].

As heart rate is inevitably linked to activity, and computation of resting HRV requires subjects to be stationary “with minimal, or unvarying, motor activity” [3], it was important to identify IBI data collected from periods of relative inactivity. In 2015 this was based on concurrent visual behavioural observations. Across the 2016 and 2017 seasons, 29 females were additionally instrumented with tri-axial accelerometers to enable us to identify periods of inactivity without the requirement for direct visual observation [38, 39]. Nine of these females were instrumented in both 2016 and 2017. Accelerometers were mounted on the crown of the head or torso, forward of the heart rate monitor transmitter unit (Fig 2A) and configured to sample at 50 or 25 Hz with a sensitivity range of ± 2 or 4 *g* depending on the year [39]. The accelerometry signal from each female was processed and transformed and summarized by a range of feature variables at a second-by-second level according to Shuert et al. [38–40]. Random forest models were trained for a simple binomial classifier of ‘Inactive’ or ‘Active’ (the latter category including all non-resting behaviours) [38–40]. Once trained, each random forest model (relating to each year of study) yielded very high classification precision (76–86%) and recall (88–98%) for the two behavioural states, regardless of accelerometer placement or sampling configuration.

## Assessment of the accuracy of IBI data produced by the Firstbeat™ system

**Fig 3:**
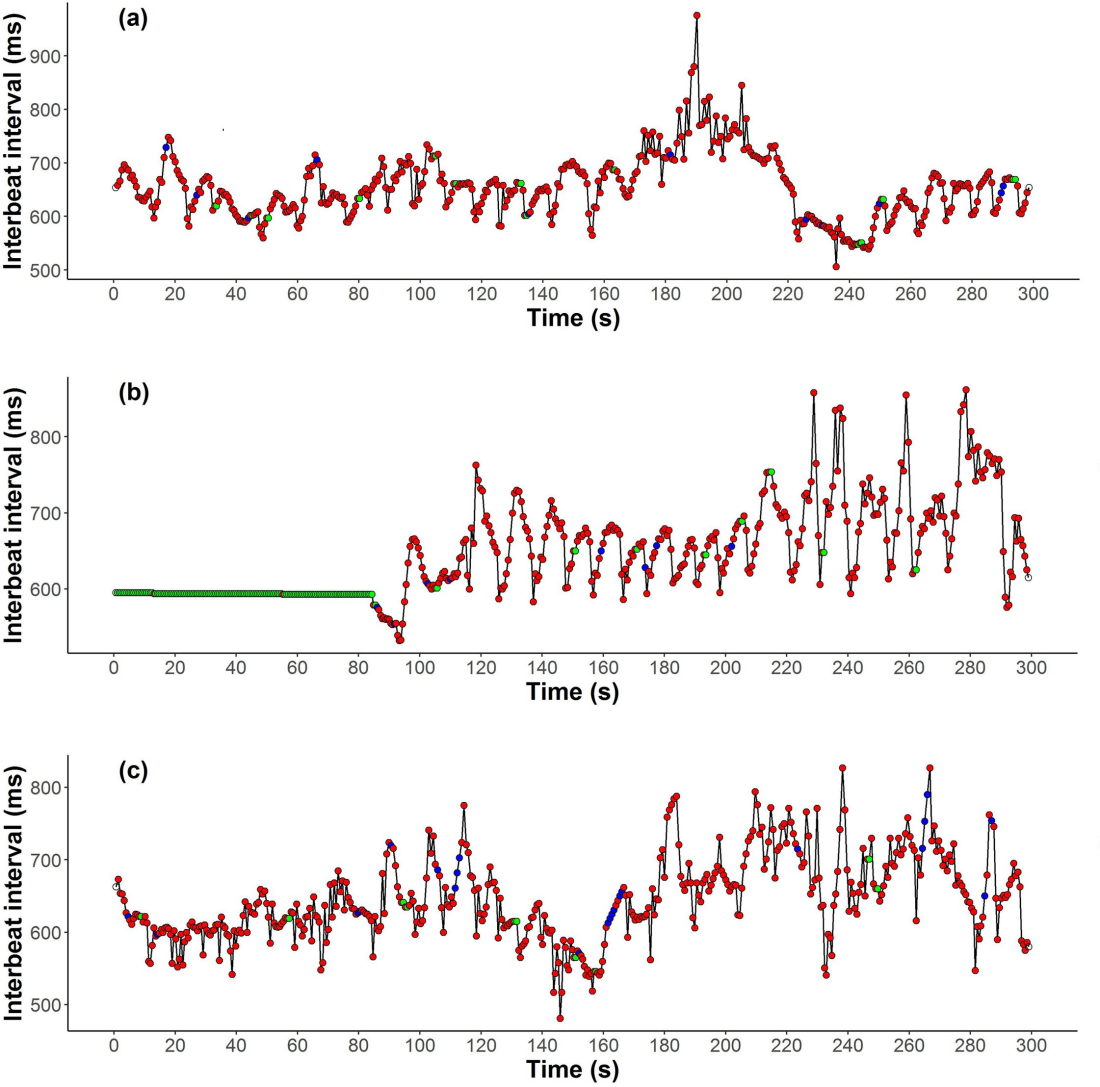
Example 300 s traces of interbeat intervals (ms), corrected for artefacts by Firstbeat™ Sports software (v.4.5.0.2), from the Firstbeat™ heart rate belts deployed on lactating grey seals. (a) A trace with less than 5% flats (green dots) and stairs (blue dots). (b) A trace with a prolonged period of flats (0 - c. 85 s). (c) A trace with sequences of stairs (c. 115 s, 165 s and 265 s). All traces also contain additional isolated flats and stairs.

The raw IBI data (in milliseconds) were first corrected for artefacts (Fig 3). We used the default options in the Firstbeat™ Sports software (v.4.5.0.2) which detects extreme values, and either deletes spurious extra beats (extreme short IBIs) or interpolates for potentially missing beats (extreme long IBIs) [41]. In order assess the accuracy of corrected IBI values generated by the Firstbeat™ system we compared them with concurrent IBI data derived from in-field ECG recordings. Prior to the recovery of telemetry equipment from the sampled seals during late-lactation captures, we used an AliveCor Heart Monitor (Model: AC-009) with the associated AliveECG Vet App (version 2.1.4, Build 17) on an iPod touch (version 8.2 (12D508)) to obtain at least 60 s of concurrent ECG [42]. Recordings were made with display settings at 50 mm/s (paper speed) and amplitudes of 20 mm/mV [42]. The AliveCor Heart Monitor electrodes were placed horizontally onto the flank of the sedated seal, immediately posterior of the left fore-flipper. If no success was had on the left flank, an ECG recording was then attempted on the right flank. The Firstbeat™ receiver was also logging IBI data simultaneously from typical recording distances. All instruments were time-synchronised (to within < 1 s) at the start of each day to ensure ability to match *post hoc* concurrent Firstbeat™ and AliveCorr readings. Only ECG traces that had clearly identifiable R peaks (from which we could extract IBI values as described below) and that had concurrent Firstbeat™ data with ≤20% flats and stairs for the duration of the ECG trace were used (examples of concurrent IBI data derived from the Firstbeat™ system and the AliveCor ECG are shown in S2 and S3 Figs in S1 File). With these criteria, we achieved a total of 16 Alive-Cor ECG traces from 12 individuals (2015 n = 10 traces | 8 individuals; 2016 n = 4 traces | 3 individuals; 2017 n = 2 traces | 2 individuals). ECG traces were saved as pdf files and converted to 300dpi jpg images using *pdf2jpg* (https://pdf2jpg.net/). R-R peak intervals (IBI values in ms) were measured from these ECG traces using Image-J v. 1.51p [43]. Measurement precision was checked by comparison of two replicate measurements of one AliveCor trace, which demonstrated that that measurements of R-R intervals were highly repeatable (S4 and S5 Figs in S1 File).

As an independent check on the corrected IBI data provided by the Firstbeat™ system, ECG data were temporally-matched with concurrent Firstbeat™ data. Agreement between these two independent measures of IBI was measured using Bland Altman analyses [12, 13, 44].

### Assessment of the causes of flats, stairs and artefacts within the Firstbeat™ IBI data

#### Identification of flats, stairs and artefacts (corrected) within the Firstbeat™ IBI data

During the artefact correction procedure, the Firstbeat™ Sports software also retains a record of which IBIs have been corrected and were therefore classified as potential artefacts by the system. As a further step, we also examined the corrected IBI data for flats and stairs. To ensure complete capture of flats and stairs, we implemented bespoke R scripts (NB, AB, ST) to identify flats (two or more consecutive identical IBI values) and stairs (sequences of more than two identical, but non-zero, differences between successive IBIs; Fig 3, S1 Fig in S1 File). Flats and stairs were not corrected but were flagged in the datasets as these types of error. We compared the identification of IBIs as artefacts by the FirstBeat^TM^ software with our additional identification of IBIs as flats or stairs.

#### Modelling the determinants of flats, stairs and artefacts (corrected) in 5min traces

Processed IBI data were then split into sequential 5 min windows [3] to allow the computation of the number of flats, stairs, and artefacts corrected by the FirstBeat^TM^ software within each 5 min trace. IBIs within the 2016 and 2017 traces were then time-matched to classified Inactive or Active behavioural states derived from the accelerometer data. These traces were used to explore potential determinants of erroneous IBIs in these traces (n = 6609 5 min traces). To do this, we fitted generalized additive mixed-effects models (GAMMs, [45]) with Gaussian distribution and identity link function (*bam*, from the ‘*mgcv’* package v1.8–24, [46]) in R version 3.5.0 [47]. We constructed separate models for the number of flats, the number of stairs, and the number of corrected artefacts by FirstBeat^TM^ software. Response variables were log-transformed (flats and stairs) or square-root transformed (corrected artefacts) to meet heteroscedasticity assumptions and address overdispersion [48]. For each model we included the following potential explanatory variables; percentage of IBI’s that were corrected for artefacts by the Firstbeat™ software (Artefacts; NB: not included in number of artefacts model), percentage of IBIs where the seal was classified as ‘active’ (Activity), day of year (Day; number of days since Jan 1), time of day (Time; proportion of 24 hr), deployment timepoint (tDeploy; days since attachment), the seal’s estimated mass on the day that the trace was recorded (Mass), and Temperature (in ^o^C, at the nearest 30 min interval to the onset of the 5 min heart rate trace) recorded from a portable Nexus Weather station (TFA Nexus, Germany) positioned at a central point on the island. We included these covariates because when examining HRV data it is important to account for individual size, physical activity, temperature effects and potential temporal patterns, all of which can influence baseline heart rates and HRV [3]. We also included tDeploy to account for potential changes in skin-electrode conductance over time (due to dissipation of electrode gel, deterioration of electrodes and/or ingress water or detritus between the skin and electrodes). Each of these variables was included as a smoothed term in our initial models as we expected nonlinear relationships between most of our independent variables and response variables. For example, Day represents the changing patterns of colonisation on the island, with a peak in seal numbers and density mid-season. Individual seal identity (ID, n_indiv_ = 29), a unique identifier for each heart rate monitor (TagID, n_tag_ = 17), and year of study (n_year_ = 2) were also included as smoothed random effects in the models [49] to account for individuals present in multiple years, tags re-used across years and interannual variation. We also included a temporal autocorrelation term, as traces recorded consecutively are more likely to exhibit similar error patterns [48, 50]. If the full model with autocorrelation indicated that the estimated degrees of freedom for any covariate was 1.00, the smoother functions for those covariates were removed and the covariates were retained as linear terms [51]. For model inference we examined all plausible alternate models with reduced combinations of explanatory variables using the R function ‘dredge’ from the Package ‘MuMIn’ [52]. We used Akaike’s information criterion (AIC) for model selection, with our ‘best’ models having the lowest AIC (ΔAIC = 0). We also retained all models within a ΔAICc ≤ 6 of the ‘best’ model within a preliminary confidence set. This confidence set was reduced further, retaining only models with a ΔAICc value lower than more complex models within which they were nested. This approach avoids retaining overly complex models but also acknowledges that the model with the lowest AICc score is not necessarily the most parsimonious model [53, 54]. For each response variable we also provide the output from the null model for comparison (models with no fixed effects and only the random effects). Greater consideration in our discussion was placed on highly significant terms (p < 0.001) due to the tendency of GAMMs to overfit, and p values > 0.001 should be treated as ambiguous as recommended in [45] and [55].

### Assessment of the effect of flats and stairs on estimates of resting HRV

To test the effect of flats and stairs on potential estimates of resting HRV we constructed simulations with varying percentages of flats or stairs in R version 3.5.0 [47]. There are multiple potential measures of sympathovagal balance that can be derived from IBI data [3] though the root mean square of successive differences (rMSSD; eqn 1) is a preferred metric for use under free-running conditions because it is impacted less by respiratory influences [56, 57] and is pragmatic in that it an easy to interpret measure of HRV [3, 6].


$$rMSSD=\sqrt{\frac{1}{N-1}\sum_{j=1}^{N-1}\Delta {IBI}_{j}^{2}}$$ (1)

Where; *N* = the length of the IBI series (1:*j*), and ΔIBI = the difference between adjacent IBI values (adapted from [24]).

We used the R package ‘RHRV’ [24] to compute rMSSD for each of the 5 min traces. We broadened the range of physiologically acceptable values for heart rate (bpm) relative to the default RHRV settings, spanning a minimum acceptable heart rate of 20bpm to a maximum of 200 bpm. Previously documented heart rates in free-living grey seals range from 4 to 120 bpm [25, 26], although extreme bradycardia of 4 bpm has only been observed during prolonged dives and was assumed unlikely to occur on land (the lowest mean heart rate among our 5 min traces was 41 bpm, with a maximum of 124 bpm).

We then selected three of the 5 min traces that had zero corrected artefacts, flats or stairs as examples of ‘perfect’ traces. We chose examples with relatively high rMSSD (107.9 ms), intermediate rMSSD (92.5 ms) and a relatively low rMSSD (69.4 ms). The effect of the proportion of flats was simulated by taking each trace in turn, randomly selecting an IBI measurement within the trace, and then changing the next IBI in the sequence to this same value to generate two consecutive identical IBI values. This process was repeated up to a maximum number of iterations equivalent to half the length of trace, so that a variety of modified traces were generated containing from one flat to traces where all IBI values were equivalent. This whole process was then repeated for 1000 runs, and rMSSD values computed for each simulated trace. A similar process was used to assess the effect of stairs, except that introduced errors involved selecting two known IBI values within the trace at random, and replacing all intervening IBIs with values that increased (or decreased) monotonically from the value of the first selected IBI to the value of the second selected IBI. Again, over 1000 iterations this created a range of simulated traces for which rMSSD values were computed, each with varying proportions of stairs from zero to 100%.

## Results

### Assessment of the accuracy of IBI data produced by the Firstbeat™ system

**Fig 4:**
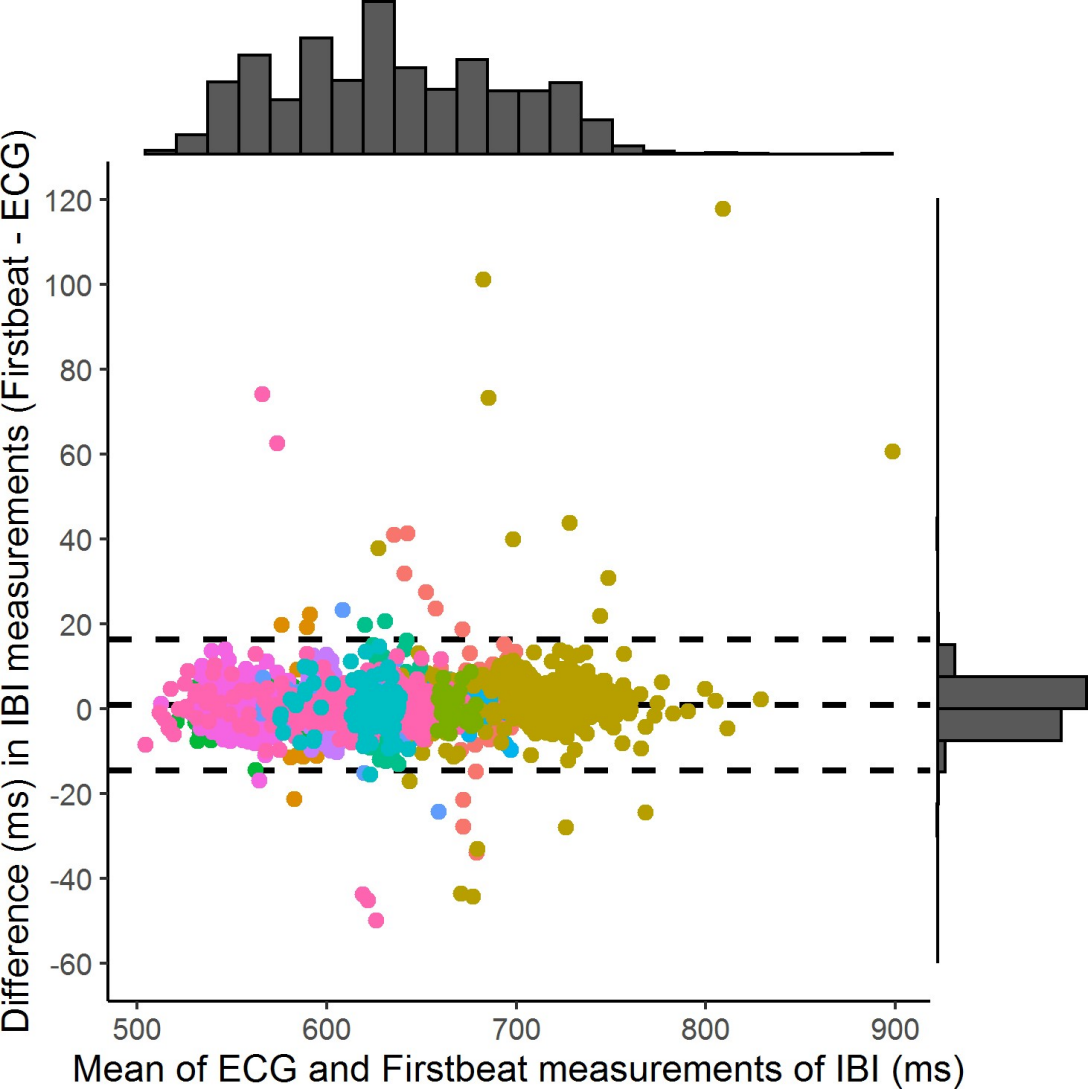
Bland-Altman plot showing limits of agreements between Firstbeat™ IBI data and AliveCor ECG measurements during recapture events (n = 16 traces | 12 individuals). Each dot represents a single IBI, and different colours indicate individual seals. Horizontal dashed lines represent mean difference (0.87 ms) and lower (-14.89 ms) and upper limits of agreement (16.61 ms). The 95% confidence intervals for the bias (mean difference, centre line) are 0.61 to 1.12 ms. The 95% confidence intervals for the lower limit of agreement (lower broken line) are -15.33 to -14.43 ms. The 95% confidence intervals for the upper limit of agreement (upper broken line) are 16.16 to 17.06 ms.

The corrected Firstbeat^TM^ IBI data showed a high degree of agreement with the AliveCor ECG measurements. With all data combined, the mean difference between Firstbeat™ IBI data and AliveCor ECG measurements was 0.87±0.16 ms (n = 16 traces | 12 individuals | 2487 IBIs). Individual traces exhibited a similar degree of agreement as determined through Bland-Altman plots and measurements of bias and limits of agreement (Fig 4). Additional plots and summary statistics for these analyses on individual traces are presented in the S6 and S7 Figs and S1 Table in S1 File.

### Comparison of FirstBeat^TM^ artefact detection with manual flats and stairs detection

Our comparison of the classification of IBIs as artefacts by the Firstbeat™ Sports software (v.4.5.0.2) with our own classification of IBIs as flats or stairs illustrates that the vast majority of flats and stairs occurred in IBIs that had been corrected by the Firstbeat™ Sports software (Table 1). 40.7% of the IBIs identified as artefacts and corrected by the Firstbeat™ Sports software were subsequently identified as flats by our algorithm (Table 1A). Only 2.2% of IBIs were identified as flats by us and not identified as artefacts by the Firstbeat™ Sports software. The vast majority (93.9%) of flats occurred in IBIs that had been corrected as artefacts by the Firstbeat™ Sports software (Table 1A). Stairs were less frequent than flats in the traces (99,242 cf. 783,625 IBIs), and only 5.5% of the IBIs identified as artefacts and corrected by the Firstbeat™ Sports software were identified as stairs by our algorithm (Table 1B). Only 0.01% of IBIs were identified as stairs by us and not identified as artefacts by the Firstbeat™ Sports software. Almost all (99.8%) stairs occurred in IBIs that had been corrected as artefacts by the Firstbeat™ Sports software (Table 1B).

**Table 1:** Matrices of all recorded IBIs (n = 3997680) showing whether they were classified as artefacts (and subsequently corrected) or not by the FirstBeat^TM^ software and whether they were categorised as flats (a), or as stairs by our bespoke processing (b). Values represent the agreement between the two algorithms. Row and column totals in *italics*.

**(a)**	Artefact	Non-Artefact	
Flat	735429	48196	*783625*
Non-Flat	1071522	2142533	*3214055*
	*1806951*	*2190729*	
**(b)**	Artefact	Non-Artefact	
Stair	99015	227	*99242*
Non-Stair	1707890	2190548	*3898438*
	*1806905*	*2190775*	

### Assessment of the causes of flats, stairs and artefacts within the Firstbeat™ IBI data

#### Determinants of flats

**Fig 5:**
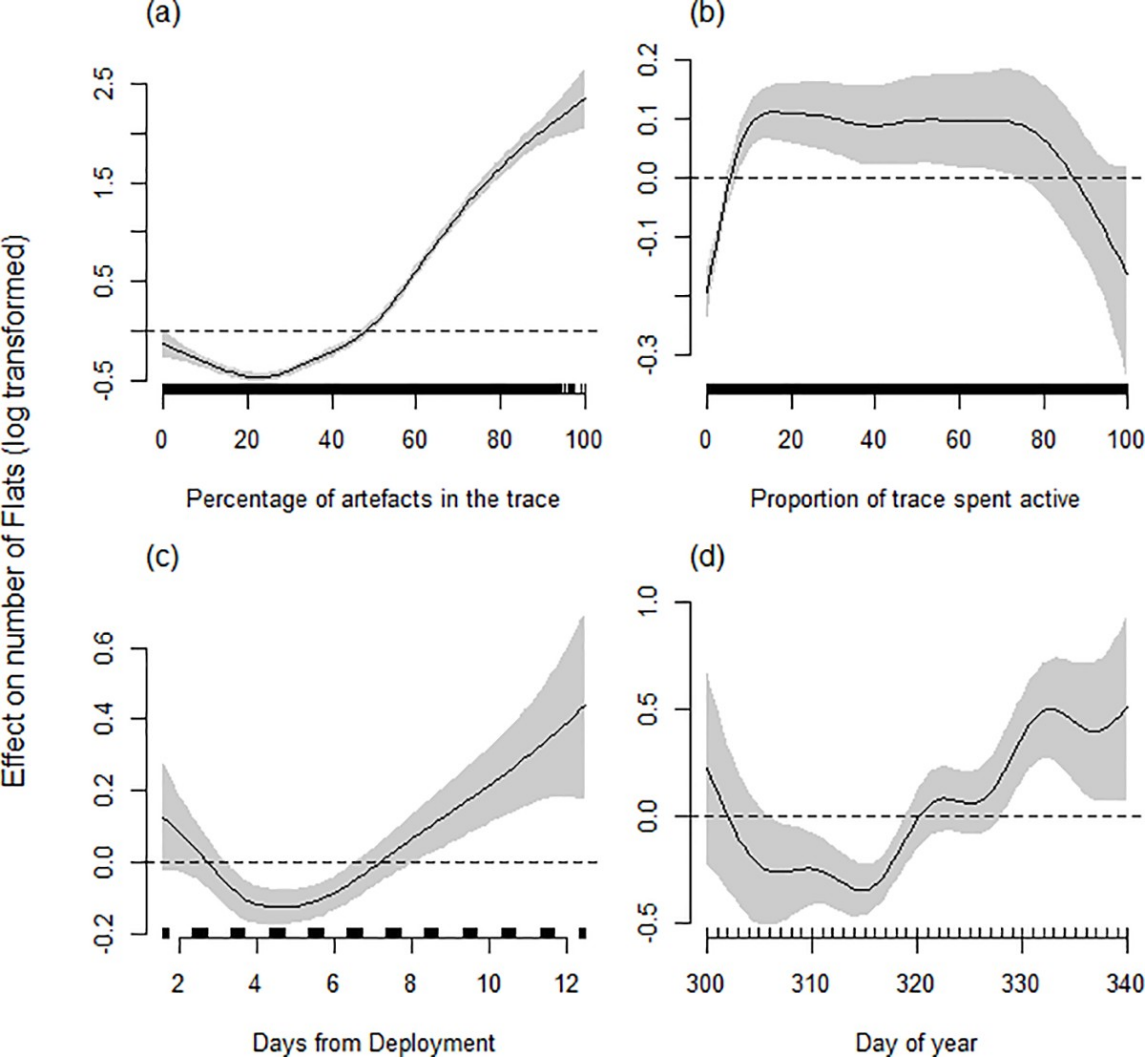
Estimated smoothing curves for GAMM describing the effect that (a) the percentage of the trace comprised of artefacts, (b) proportion of activity, (c) deployment time and (d) day of year have on the number of flats in 5 min IBI traces for female grey seals. The smoothing curve is indicated by the solid black line with approximate 95% confidence intervals represented by grey shading. On the y-axis, 0 indicates no effect of the covariate, while positive values indicate positive correlation and negative values indicate negative correlation. The effect, relative to the mean number of stairs (dashed line), for a particular value of a covariate can be obtained as the natural antilogarithm of the y-axis value for the smoothing curve. The sampling spread of HR traces across each covariate scale is indicated by above the x-axis.

The best model accounting for the number of flats in a trace included the linear terms Time and Temperature and the smooth terms; Artefacts, Activity, tDeploy and Day (Table 2). The number of flats increased with time as a proportion of 24 hr. The total number of artefacts also had a noticeable effect on the number of flats in a trace (Fig 5A). As the percentage of the trace that is an artefact increases beyond 50%, the number of flats increased steeply. The number of flats also increased with activity up to approximately 20% of the trace being classified as active; further increases in activity had no additional effect on the number of flats (Fig 5B). There also was a general pattern of more flats in traces recorded later in a deployment, and later in the season compared to earlier in the season (Fig 5C and 5D). The random smooth terms for individual ID and heart rate monitor ID were retained, though the effect of the monitor ID was much smaller than individual ID (Table 2). Year was excluded from the model during model selection procedures.

**Table 2:** Estimated degrees of freedom (edf) for smoothed terms, estimate and standard error for linear terms from the ‘best’ model predicting number of flats (log transformed) in traces.

Explanatory variable				
**A. Linear terms**	**Estimate**	**Std. Error**	**t-value**	**P value**
Time	0.72	0.13	5.39	**< 0.0001**
Temperature	0.013	0.0065	1.93	0.054
**B. Smooth terms**	**edf**		**F-value**	**p-value**
Day	15.38		5.40	**< 0.0001**
tDeploy	4.53		8.81	**< 0.0001**
Activity	5.91		12.69	**< 0.0001**
Artefacts	6.43		304.77	**< 0.0001**
**Random smooth effects**				
ID	23.88		58.48	**< 0.0001**
Heart rate monitor ID	6.69		39.34	0.0095

*Rho* = 0.18. Comparison with null model; ΔAIC = 2130.0. Deviance explained = 39.6%. Model details: n(traces) = 6609, n(individuals) = 29, n(heart rate monitor ID) = 17. Highly significant terms (p < 0.001) are in bold [55]. See S2 Table in S1 File for details of the model confidence set.

Basis dimension (k) = 9 for all smooth terms except for Day (k = 24)

#### Determinants of stairs

**Fig 6:**
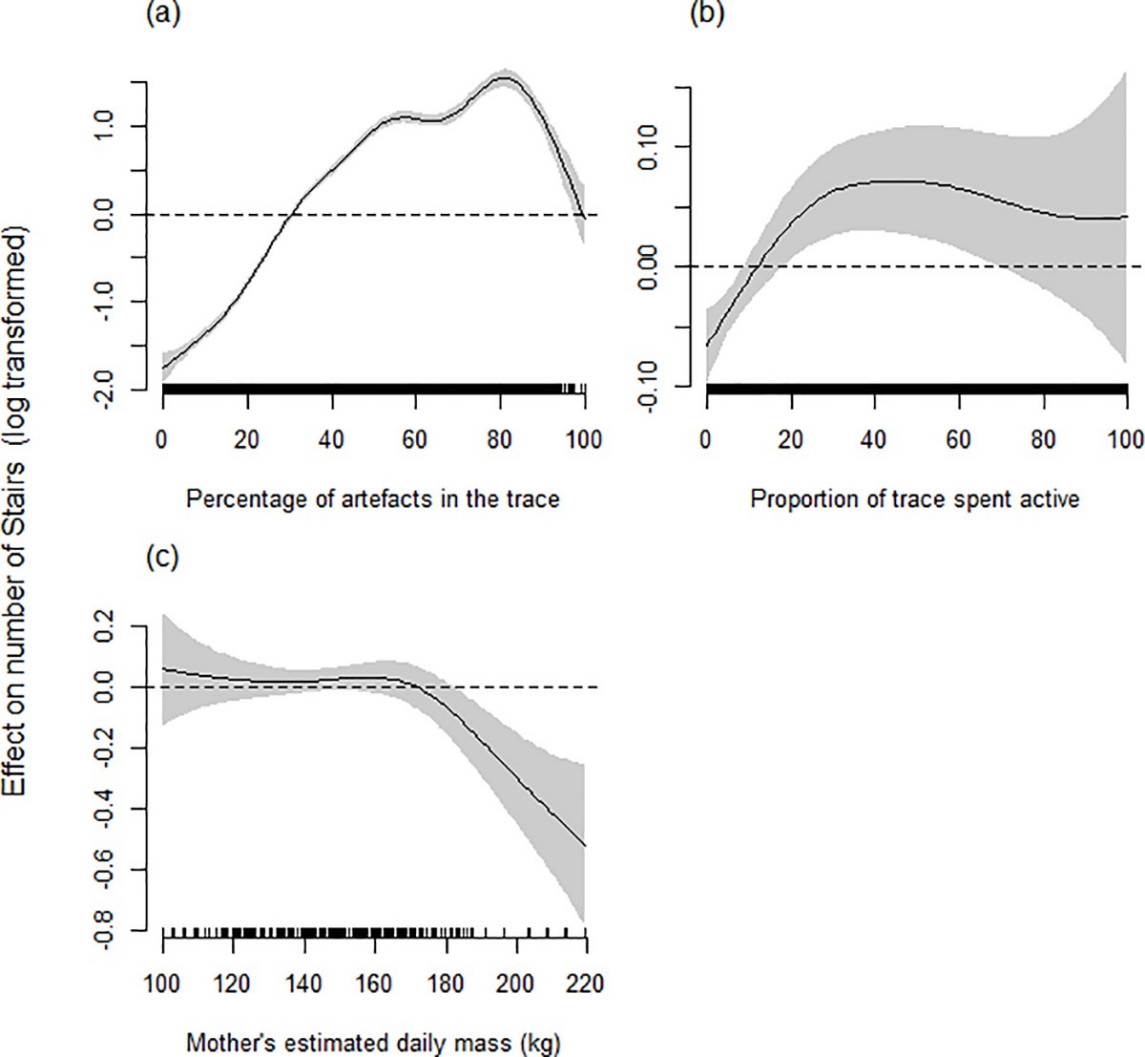
Estimated smoothing curves for GAMM describing the effect that (a) the percentage of artefacts, (b) proportion of activity and (c) temperature have on the number of stairs in 5 min IBI traces for female grey seals. The smoothing curve is indicated by the solid black line with approximate 95% confidence intervals represented by grey shading. On the y-axis, 0 indicates no effect of the covariate, while positive values indicate positive correlation and negative values indicate negative correlation. The effect, relative to the mean number of stairs (dashed line), for a particular value of a covariate can be obtained as the natural antilogarithm of the y-axis value for the smoothing curve. The sampling spread of HR traces across each covariate scale is indicated by above the x-axis.

The best model accounting for the number of stairs (log transformed) in a trace (Table 3) included the smoothed terms Artefacts, Activity, and Temperature. Stairs increased with increasing artefacts until approximately 80% of the trace comprised artefacts, after which the number of stairs declined (Fig 6A). Activity exhibited a similar pattern to that seen in the analysis of flats. The number of stairs increased up to approximately 30% activity and then plateaued (Fig 6B). The number of stairs in a trace was greatest at intermediate temperatures (Fig 6C), though with wide confidence intervals at the extremes of the observed temperature range. The only random smooth term retained was individual ID (Table 3); heart rate monitor ID and Year were excluded from the model during model selection procedures.

**Table 3:** Estimated degrees of freedom (edf) for smoothed terms, estimate and standard error for linear terms from the ‘best’ model predicting number of stairs (log transformed) in traces.

Explanatory variable			
**Smooth terms**	**edf**	**F-value**	**p-value**
Activity	3.08	5.74	**< 0.0001**
Artefacts	13.19	225.07	**< 0.0001**
Temperature	1.55	0.78	0.52
**Random smooth effects**			
ID	17.11	2.14	**< 0.0001**

*Rho* = 0.05. Comparison with null model; ΔAIC = 3533.4. Deviance explained = 56.1%. Model details: n(traces) = 6609, n(individuals) = 29. Highly significant terms (p < 0.001) are in bold [55]. See S2 Table in S1 File for details of the model confidence set.

Basis dimension (k) = 9 for all smooth terms except for Artefacts (k = 24).

#### Determinants of corrected artefacts

**Fig 7:**
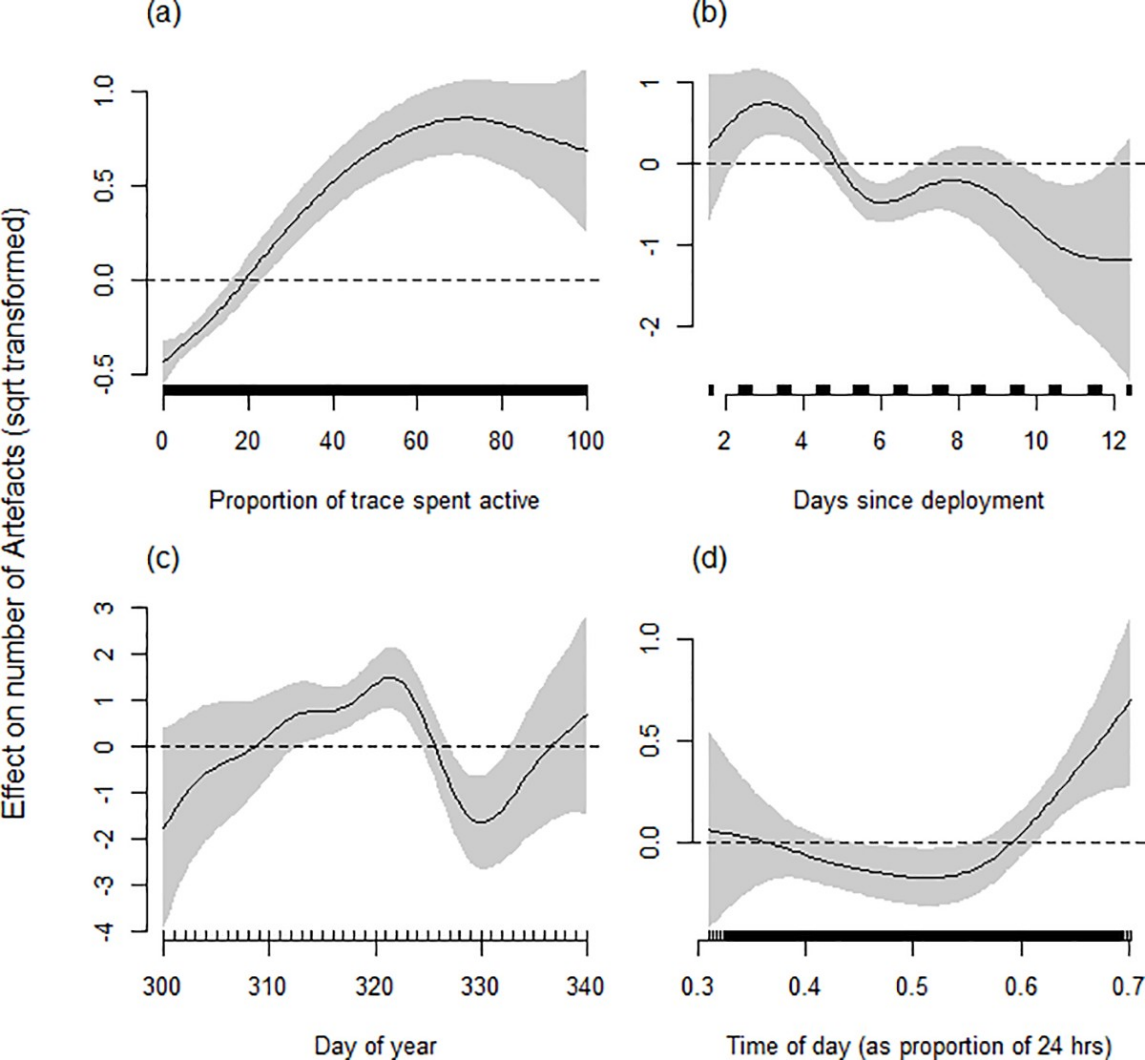
Estimated smoothing curves for GAMM describing the effect that (a) proportion of activity, (b) deployment time, (c) day of year and (d) time of day have on the number of artefacts identified by the Firstbeat^TM^ software in 5 min IBI traces for female grey seals. The smoothing curve is indicated by the solid black line with approximate 95% confidence intervals represented by grey shading. On the Y axis, 0 indicates no effect of the covariate, while positive values indicate positive correlation and negative values indicate negative correlation. The effect, relative to the mean number of stairs (dashed line), for a particular value of a covariate can be obtained as the natural antilogarithm of the y-axis value for the smoothing curve. The sampling spread of HR traces across each covariate scale is indicated by above the x-axis.

The best model accounting for the number of Firstbeat™ corrected artefacts (square root transformed) in a trace included the smoothed terms Activity, tDeploy, Day, Time, Mass and Temperature (Table 4). The number of artefacts in traces generally increased with activity (Fig 7A), though again the modelled relationship tended to plateau around 70–80% activity. In relation to tDeploy, the number artefacts showed an initial rise between days 3 and 5, followed by a general decline over the remainder of the deployment period (Fig 7B). Day showed an irregular relationship with number of corrected artefacts (Fig 7C), with a tendency towards more artefacts in traces during the mid-season period. Time had limited effect on number artefacts other than towards the end of the daylight period where there was a relatively small increase in artefacts in traces (Fig 7D). The random smooth terms for individual ID and heart rate monitor ID were retained (Table 4). Year was excluded from the model during model selection procedures.

**Table 4:** Estimated degrees of freedom (edf) for smoothed terms, estimate and standard error for linear terms from the ‘best’ model predicting number of corrected Artefacts (square root transformed) in traces.

Explanatory variable			
**Smooth terms**	**edf**	**F-value**	**p-value**
Day	12.7	4.04	**<0.0001**
Time	2.90	4.89	0.0013
tDeploy	5.55	4.03	**0.00033**
Activity	3.28	31.14	**<0.0001**
Mass	2.94	1.30	0.31
Temperature	2.96	1.18	0.25
**Random smooth effects**			
ID	22.45	233.86	**<0.0001**
Heart rate monitor ID	9.55	339.20	**0.00016**

*Rho* = 0.42. Comparison with null model; ΔAIC = 225.4. Deviance explained = 41.3%. Model details: n(traces) = 6609, n(individuals) = 29, n(heart rate monitor ID) = 17. Highly significant terms (p < 0.001) are in bold [55]. See S2 Table in S1 File for details of the model confidence set.

Basis dimension (k) = 9 for all smooth terms except for Day (k = 24)

### Assessment of the effect of flats and stairs on estimates of resting HRV

**Fig 8:**
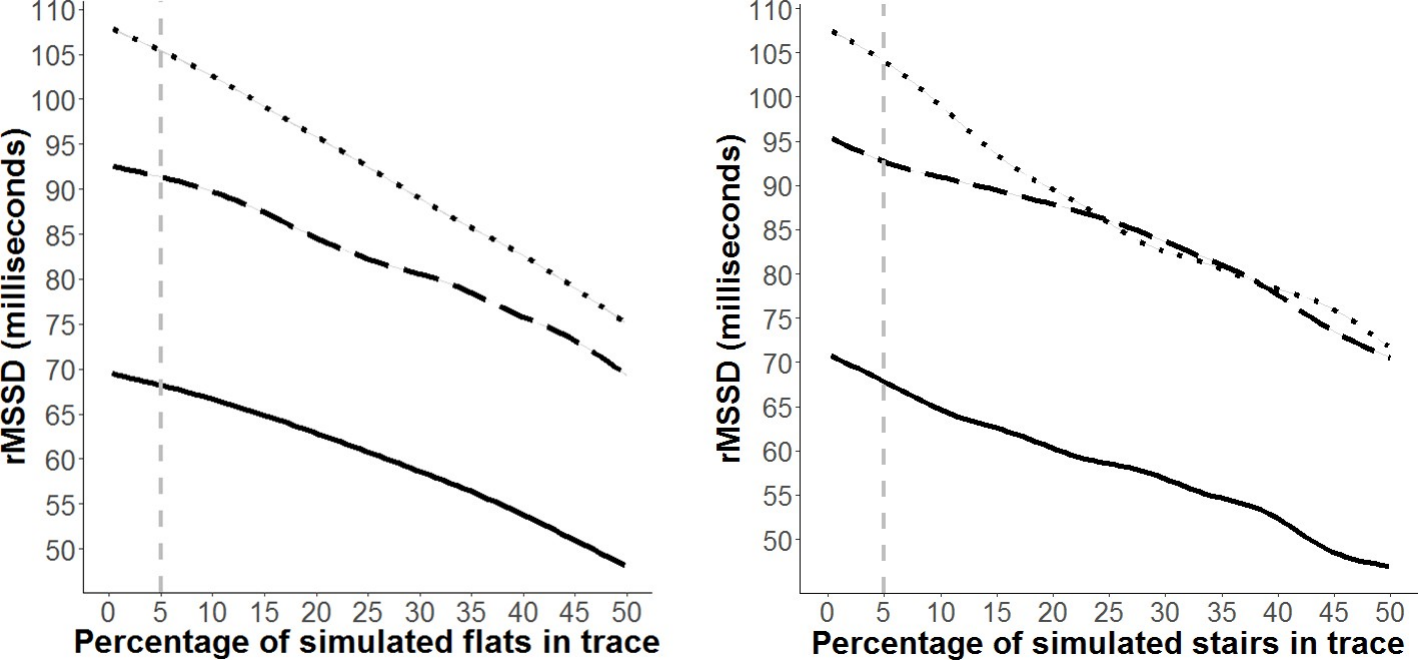
Output of n = 1000 simulations for increasing proportion of flats (a) and stairs (b) in IBI traces with high (dotted curve), moderate (dashed curve) and low (solid curve) resting rMSSD estimates. Proportion of flats and stairs shown to a maximum of 50% of the trace, rMSSD estimates diminish to zero at 100% flats or stairs in all traces. Vertical grey dashed line indicates the 5% flats or stairs for reference. The curves do have 95% confidence intervals plotted, but these are narrower than the width of the plotting line, so are not visible.

Simulations of increasing proportions of flats or stairs in a trace showed that even at small proportions (≤ 5%) flats and stairs tend to lead to a decline in rMSSD (Fig 8). Within this range, the effect was essentially linear; 5% flats in the trace resulted in a reduction of rMSSD by 2 ms for the high trace, and 1 ms for the moderate and low traces (Fig 8). The presence of stairs tended to cause a more rapid decline in rMSSD; with 5% stairs reducing the rMSSD estimate by 4 ms for the high trace, and 2–3 ms for the moderate and low traces (Fig 8).

## Discussion

Here, we have described and assessed a robust, externally mounted heart rate monitor for use in wild mammals, that delivers millisecond precision measures for IBI’s, allowing computation of key physiological parameters, in particular, measures of resting HRV when coupled with information on individual activity [38, 39, 58]. Our system allows for collection of IBI data on many individuals concurrently over prolonged time-periods. Even with quite stringent filtering procedures to discard traces with excessive artefacts and unwanted levels of animal activity, we were able to retain a useful sample size of individuals, and large number of traces per individual. We assessed the accuracy of the IBI data transmitted by the modified Firstbeat™ heart rate belts on a subset of individuals during recapture events. These tests showed very close agreement between the corrected Firstbeat™ IBI measurements and those derived independently from in-field ECG traces, with < 1 ms difference in measurements of IBIs ranging from approximately 500 to 900 ms duration, equating to approximately 0.002% difference at most.

One of the key objectives of this effort was to develop a system capable of remotely recording IBI data with millisecond precision suitable for computing resting HRV (and heart rate) metrics, simultaneously from multiple freely moving wild animals that are not restrained or constrained by laboratory settings. However, this comes with a trade-off, as animal activity increased the number of artefacts identified by the Firstbeat™ software, and the number of flats and stairs. Physical activity is known to cause artefacts due to noise from muscle action potentials [3, 5]. In our study, higher levels of activity however had little additional effect. This may be a product of the coarse classification of activity we have used (active vs. inactive), as activity includes a wide range of behaviours from minor movements (such as vigilance behaviours) to intense aggressive interactions [27, 31, 38]. Similarly, day of year and (to a lesser extent) time of day also contributed to explaining variation in the occurrence of artefacts, with artefacts more prevalent during the mid-season period and at the end of the daylight period. These temporal patterns are likely mediated though changing seal behaviour (as opposed to ‘activity’ per se) across the breeding season and diurnally [3]. Artefacts may be more commonly associated with specific types of activity than others (e.g. locomotion will likely generate more artefacts than head movements associated with vigilance behaviours), however, our binary classification of activity would not be able to resolve this.

The presence of flats and stairs in our traces were highly associated with the artefact correction procedures in the Firstbeat^TM^ Sports software, especially where a large proportion of a trace comprised corrected IBIs. Essentially, the Firstbeat™ software detects extreme IBI values, and either deletes spurious extra beats, or interpolates for potentially missing beats [41]. When there are multiple missing beats (e.g. during a period of poor or obscured transmission), the software automatically interpolates the signal, resulting in a sequence of identical IBI values (flats) or monotonically changing values (stairs). The relationship between the percentage of a trace comprised of corrected artefacts and the occurrence of flats was not linear. In fact, the occurrence of flats diminishes in traces with approximately 10–40% of corrected artefacts. Lower percentages of artefacts are more likely to be single missing beats spread across the trace, in which case interpolation will not necessarily result in a detectable flat or stair (using our criteria for these types of error), and the Firstbeat^TM^ Sports software has more informative adjacent IBIs to enable reasonable correction of extreme values. At higher percentages of artefacts, it is inevitable that a greater proportion of missing beats will be adjacent, resulting in interpolation generating flats (or stairs). For stairs, our results showed that at extremely high levels of corrected artefacts (over ca. 80%) the number of stairs declines. Stairs occur where the software interpolates missing beats lying between two beats with differing values. At extremely high levels of corrected artefacts, there will be few ‘valid’ data points, and interpolation is more likely to be in the form of flats than stairs (noticeably the occurrence of flats continues increasing up to 100% of corrected artefacts). Additional flats (or stairs) will be generated in traces via the artefact correction procedure when line-of-sight signal transmission is interrupted, even momentarily. In our system, where seal morphology necessitated placement of the transmitter dorsally, this included occasions where a seal rolled onto their back. Furthermore, due to the generally supine position of seals, even relatively small geographical features (e.g. rocks, walls) would obscure transmission once seals moved out of line-of-sight of the receiver.

The presence of flats and stairs did lead to a reduction in IBI variability and therefore rMSSD values in our simulations. For traces with up to 5% of flats or stairs the impact was minimal and linear; however, the effect was greater for individuals with higher resting HRV, which consequently has the effect of making inter-individual comparisons more conservative. These findings are in line with those identified in previous studies [3, 8] and can be used to inform criteria for selecting traces deemed reasonable for computing resting HRV estimates for individual seals. Retaining traces with up to 5% flats or stairs would have minimal impact on estimates of rMSSD but allow researchers to maximise the number of useable traces. In addition, since the effect of the percentage of flats or stairs on HRV in our simulations was relatively linear, at least in this range of 0–5% flats or stairs, one could readily implement a correction for rMSSD values derived from our 5 min traces based on the remaining percentage of flats (0–5%) in a trace.

Finally, in all models, individual identity had a highly significant effect, suggesting individual differences in the occurrence of flats, stairs and artefacts. There are potentially many influences at play here, such as individual differences in location and micro-habitat, especially with respect to access to water [27, 28, 59], behaviour patterns [60, 61], or even the possibility of cognitive control of heart rate [62] that may influence the occurrence of flats and stairs. Heart rate monitor tag ID was also retained in the best models for flats and artefacts, but we were unfortunately unable to conduct a crossed experimental design to thoroughly disentangle the effects of individual ID and tag ID.

### Telemetry system advantages and limitations

The modified Firstbeat™ system we deployed here has several notable advantages for exploring physiological questions *in situ* on wild animals. Overall, the Firstbeat™ system proved to be a reliable and minimally invasive method for gathering large amounts of IBI data on multiple individuals simultaneously, while the subjects are engaged in natural behaviour over multiple days in natural conditions. The devices proved robust to the conditions of the colony, withstanding being rolled on rocks, dragged through mud and water, and surviving aggressive encounters between individuals.

Less exposed implantable heart rate data loggers have been used in other studies that can either record data internally [18] or communicate with an externally mounted transmitter [6]. However, such systems require the re-acquisition of the logger in order to retrieve data, necessitating recapture of the individuals involved and multiple surgical procedures. An advantage of the Firstbeat^TM^ system is that it provides greater certainty of acquiring some data on all study individuals, even if they ultimately prove impossible to recapture, because the system continually transmits IBI data to a remote receiver. In our study, we did recapture animals, but only at the end of the planned deployments (end of lactation) to retrieve the transmitters for future use. Furthermore, implantable heart rate data loggers suffer the drawback of typically summarising heart rate over discrete time periods (e.g. every 2–30 s) or only estimating instantaneous heart rate to the nearest 10–50 ms, rendering them unsuitable for conducting HRV analyses [8].

Another distinct advantage of the Firstbeat™ system, compared to other split transmitter-receiver systems, such as the Polar® RS800CX system (which requires each receiver to be paired to a single transmitter [8]), is that the Firstbeat™ Receiver can monitor multiple individuals simultaneously. The Firstbeat™ system can also log data from transmitters located at distances of up to 200 m from the receiver, compared to the very limited range (c. 20–50 m) of the RS800CX system [8]. This decreases the potential for disturbance associated with multiple approaches and the placement of receivers in close proximity to study animals. Whilst 200 m is a substantial recording range, the system does require a study species that has a restricted range, at least for a period of time, such as seals confined to a breeding colony, or a set-up that enables mobility of receiving stations (e.g. following a migrating herd of ungulates). For example, in our study, we were limited to a single Firstbeat™ Receiver 30, and we could not achieve line of sight on all instrumented seals simultaneously due to the to the rugged terrain and patchy aggregation of instrumented seals. However, additional infrastructure (e.g. more receivers coupled to permanent base-stations) would extend the capability of this system and permit continuous data collection (overnight) on a larger number of individuals. Firstbeat™ also produce a Team-receiver capable of recording up to 80 transmitters simultaneously. In more open terrain, such a set-up could achieve very high sample sizes. Further enhancements of our system could be made by improving battery life of the transmitter unit for more prolonged deployment. However, this would also require an improvement in electrode design. Our results show that the performance of our electrodes declined over time (although adequate for our 13 d deployments). The use of sub-dermal electrodes (where ethical considerations permit) would assist in reducing artefacts due to poor skin-electrode conductance and enable prolonged deployment.

While our focus here has been on gathering millisecond precision IBI measurements to provide resting HRV measures [3, 37, 38], the Firstbeat^TM^ system is equally suitable for acquiring heart rate measures from free ranging animals. Basic heart rate metrics are widely used in studies of wild animal physiology, behaviour and energetics (e.g. [14, 15, 61, 63]) and so the devices we describe here have potentially wide applicability across these research efforts. With the potential for continuous recording of IBI data from multiple individuals simultaneously, the system we describe here could provide extensive data to examine circadian and seasonal cycles in heart rate, or changes in heart rate with activity and context (e.g. social context) or in responses to stressors, including anthropogenic stressors, in free-ranging species [14, 15, 61–64] while providing sufficient data to allow stringent error filtering for deriving estimates of heart rate and resting HRV. Further, the results presented here provide researchers with a pragmatic approach for filtering acquired data that enable extraction of a robust estimates of resting HRV (and heart rate) from wild animals in natural settings, while balancing the requirement for relatively error free IBI data.

## Supporting information

S1 File (DOCX)

S1 Data (TXT)

S2 Data (TXT)
