# Supplementary material for: An external telemetry system for recording resting heart rate variability and heart rate in free-ranging large wild mammals
Source: PLoS One. 2021 Jun 4;16(6):e0252013. doi: 10.1371/journal.pone.0252013 (PMC8177659; doi:10.1371/journal.pone.0252013)
Supplement: S1 File — (DOCX) [file pone.0252013.s001.docx]

**An external telemetry system for recording resting heart rate variability and heart rate in free-ranging large wild mammals**

Sean D. Twiss ^1*¶^, Naomi Brannan^1,#a¶^, Courtney R. Shuert ^1,#b¶^, Amanda M. Bishop ^1,#c¶^, Patrick. P. Pomeroy^2¶^, Simon Moss^2¶^

^1^ Department of Biosciences, Durham University, Durham, United Kingdom

^2^ Sea Mammal Research Unit, Scottish Oceans Institute, University of St. Andrews, St. Andrews, United Kingdom

^#a^ Current Address: Southeast Asia Marine Mammal Research (SEAMAR),

17/F Lippo Centre Tower 1, 89 Queensway, Admiralty, Hong Kong Special Administrative Region

^#b^ Current Address: Department of Integrative Biology, University of Windsor, Ontario, Canada

^#c^ Current Address: Water and Environmental Research Center, University of Alaska Fairbanks, Fairbanks, Alaska

* Corresponding author

E-mail: [s.d.twiss@durham.ac.uk](mailto:s.d.twiss@durham.ac.uk) (SDT)

¶These authors contributed equally to this work.

**
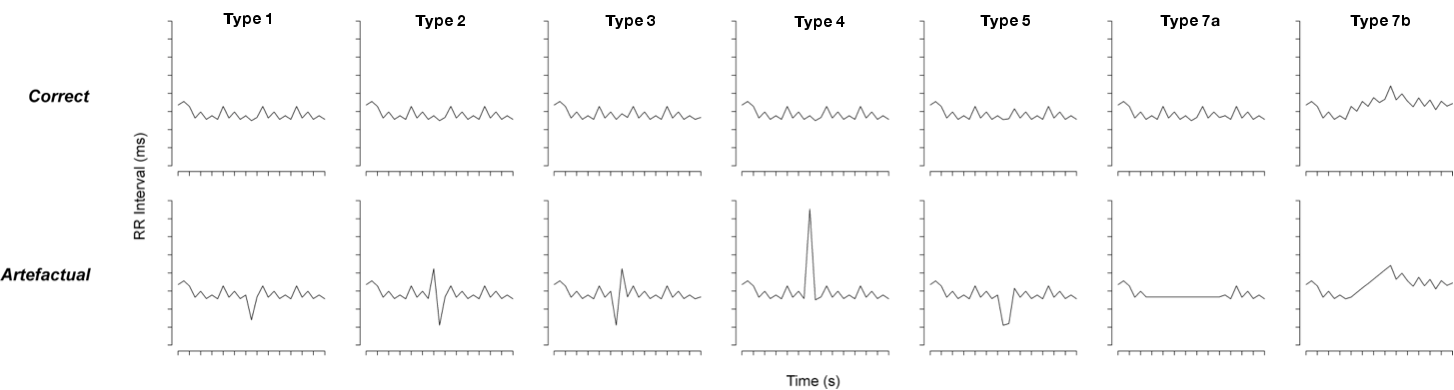
**

**S1 Fig.** **Common artefacts in IBI data derived from externally mounted heart rate monitors.** Type 1 to 5 artefacts exhibit anomalous spikes and troughs and are categorised as “Peaks”. Type 1 artefacts are single point discrepancies that can be either positive or negative. Type 2 artefacts are characterised by a long IBI (a spike) followed by a compensatory short IBI (a trough). Type 3 artefacts are similar to Type 2 artefacts, except they are characterised by a short IBI followed by a compensatory long IBI. Type 4 artefacts are characterised by 2–3 extremely long IBIS, often two or three times longer than the surrounding IBIS. Type 5 artefacts are characterised by a flat-bottomed trough, consisting of two short IBIS. Type 7a and Type 7b artefacts are characterised by invariable sequences that differ in the number of IBIs recorded. are what we term as flats and stairs respectively. Type 7a artefacts occur when an IBI is the same as the interval preceding it (flats). Type 7b artefacts occur when the difference between successive intervals is the same (stairs). *Adapted from* Marchant-Forde *et al.* (2004) *and* Jonckheer-Sheehy *et al.* (2012).


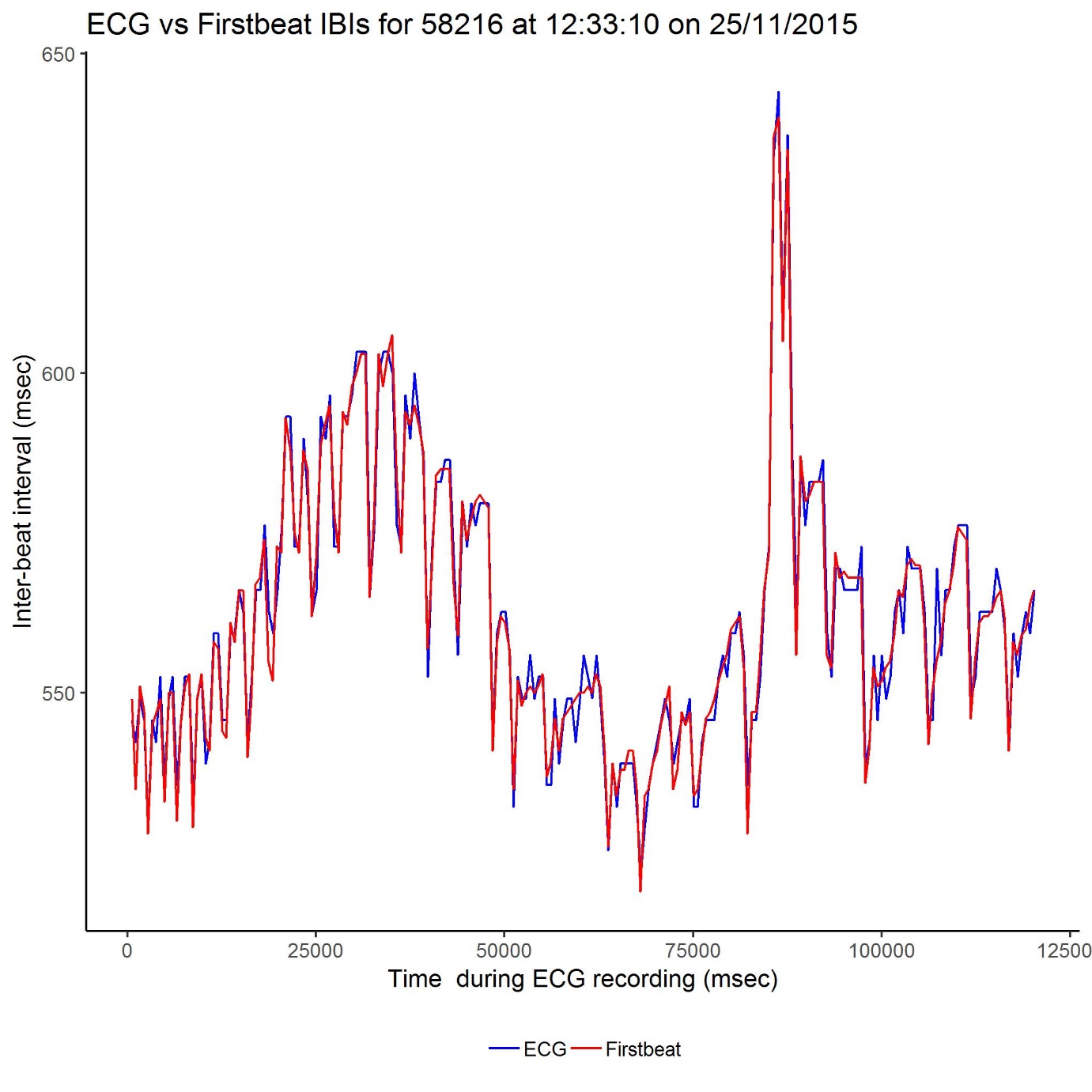


**S2 Fig. Concurrent IBI data derived from the Firstbeat™ system (red) and the AliveCor ECG (blue) for female ID17 at 12:33:10 GMT on 25/11/2015.** Illustration of the high degree of agreement between IBI values derived from the in-field recordings by the Firstbeat™ system and the ECG recordings.


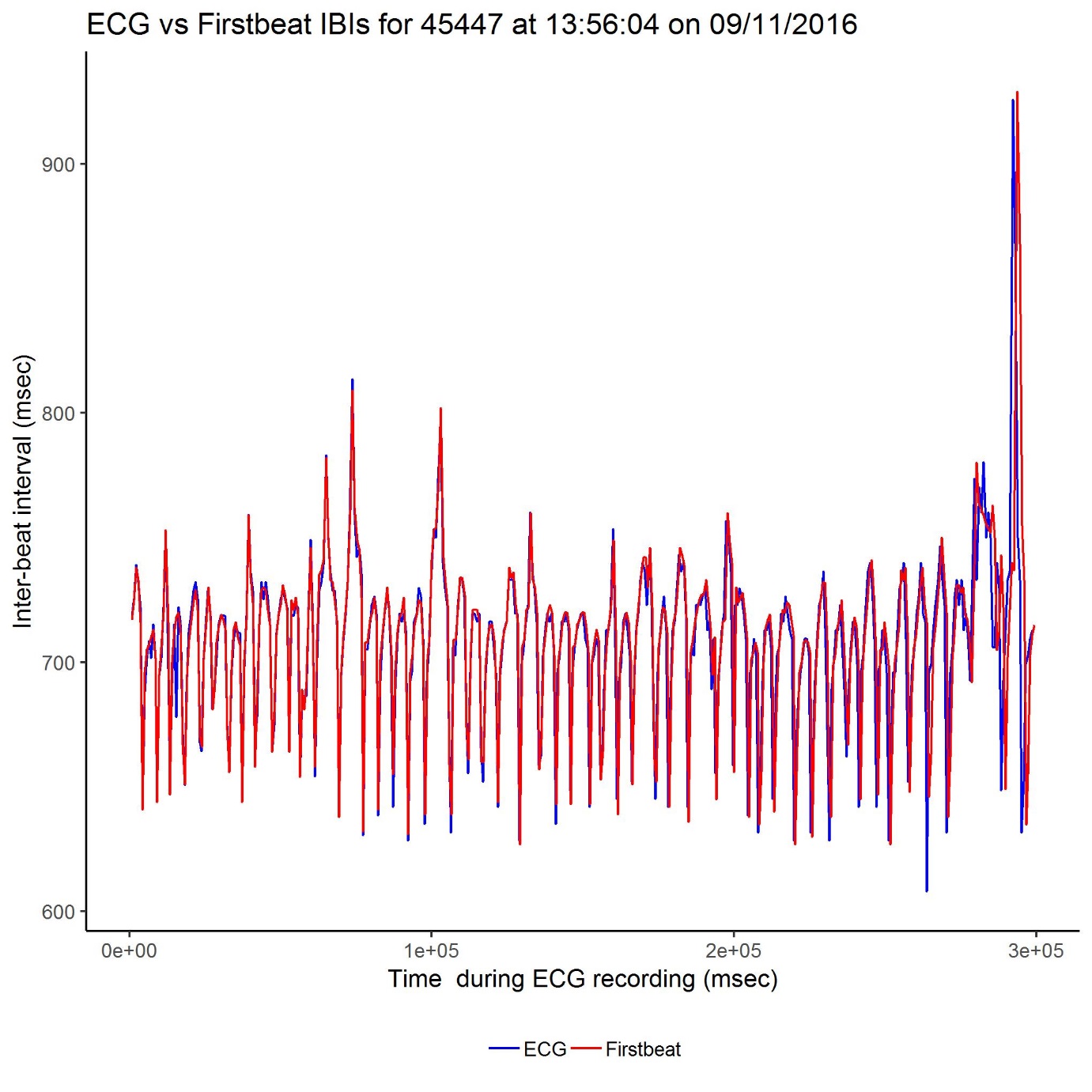


**S3 Fig. Concurrent IBI data derived from the Firstbeat™ system (red) and the AliveCor ECG (blue) for female ID3 at 13:56:04 GMT on 09/11/2016.** Illustration of the high degree of agreement between IBI values derived from the in-field recordings by the Firstbeat™ system and the ECG recordings.


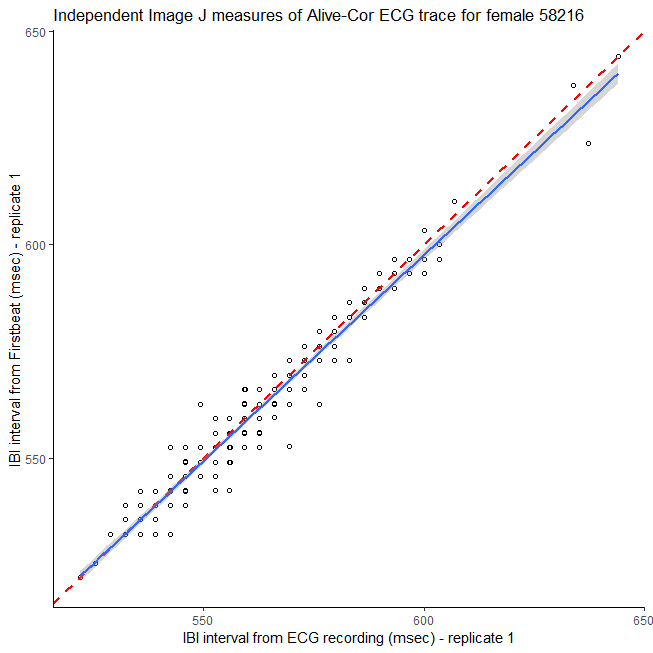


**S4 Fig. Comparison of the two replicate measurements of R-R peak intervals from one trace (comprising 214 heart beats).** The observer (SDT) repeated the extraction of R-R peak intervals from one trace on two occasions separated by 30 days. This was to test the attainable precision of measuring R-R peak intervals using Image-J v. 1.51p (<https://imagej.nih.gov/ij/>; Schneider et al. 2012). Comparison of the two replicate measurements of the trace (comprising 214 heart beats) indicated that measurements of R-R intervals were highly repeatable (ICC3k = 0.99, F_213,213_ = 91, p < 0.0001, lower 95 % CI = 0.98, upper 95 % CI = 0.99). The mean difference between replicates was 0.96 ± 0.29 ms, suggesting that there was typically a 1 ms difference between repeat measures of the same R-R interval (range of RR interval values in the sample trace: 522.034 - 644.068 ms).


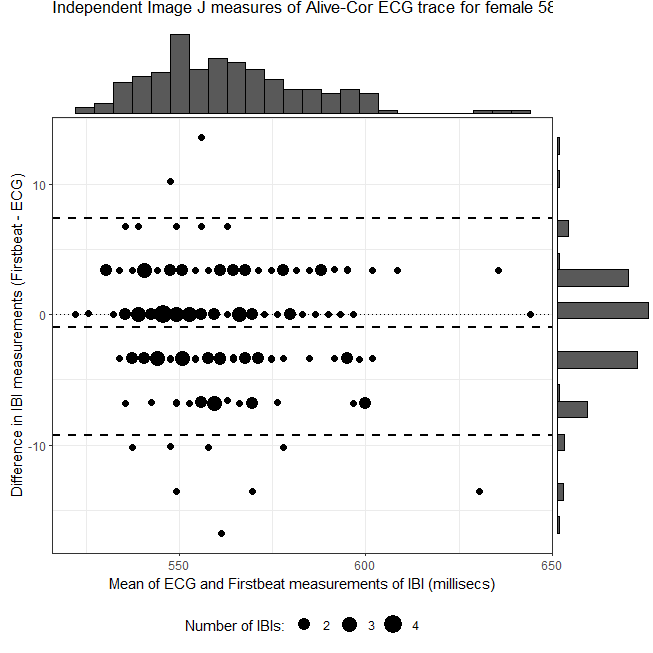


**S5 Fig. Bland-Altman plot showing limits of agreements between the two replicate measurements of R-R peak intervals from one trace (comprising 214 heart beats).** Horizontal dashed lines represent mean difference and lower and upper limits of agreement.


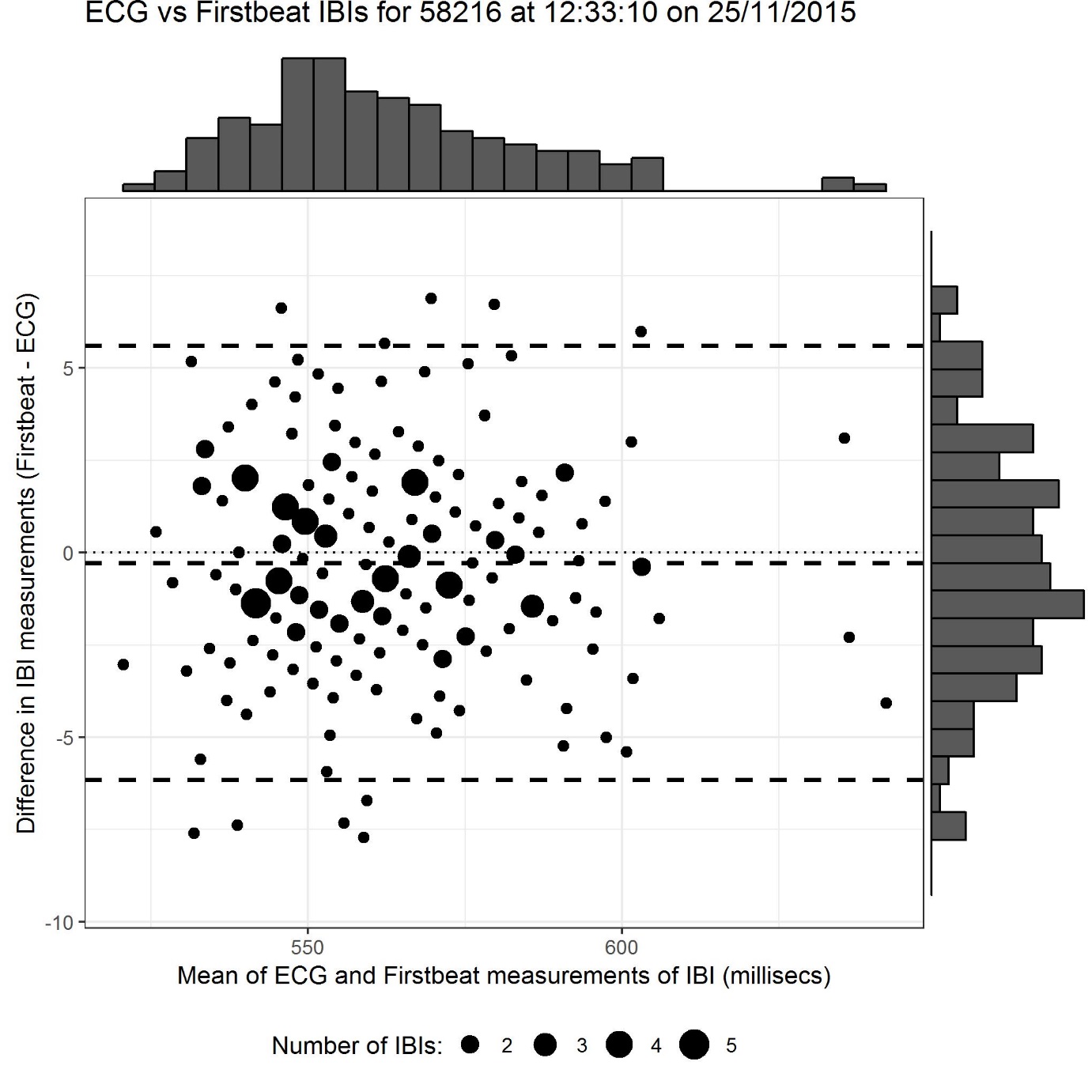


**S6 Fig. Bland-Altman plot showing limits of agreements between concurrent IBI data derived from the Firstbeat™ system and the AliveCor ECG for female ID17 at 12:33:10 GMT on 25/11/2015.**


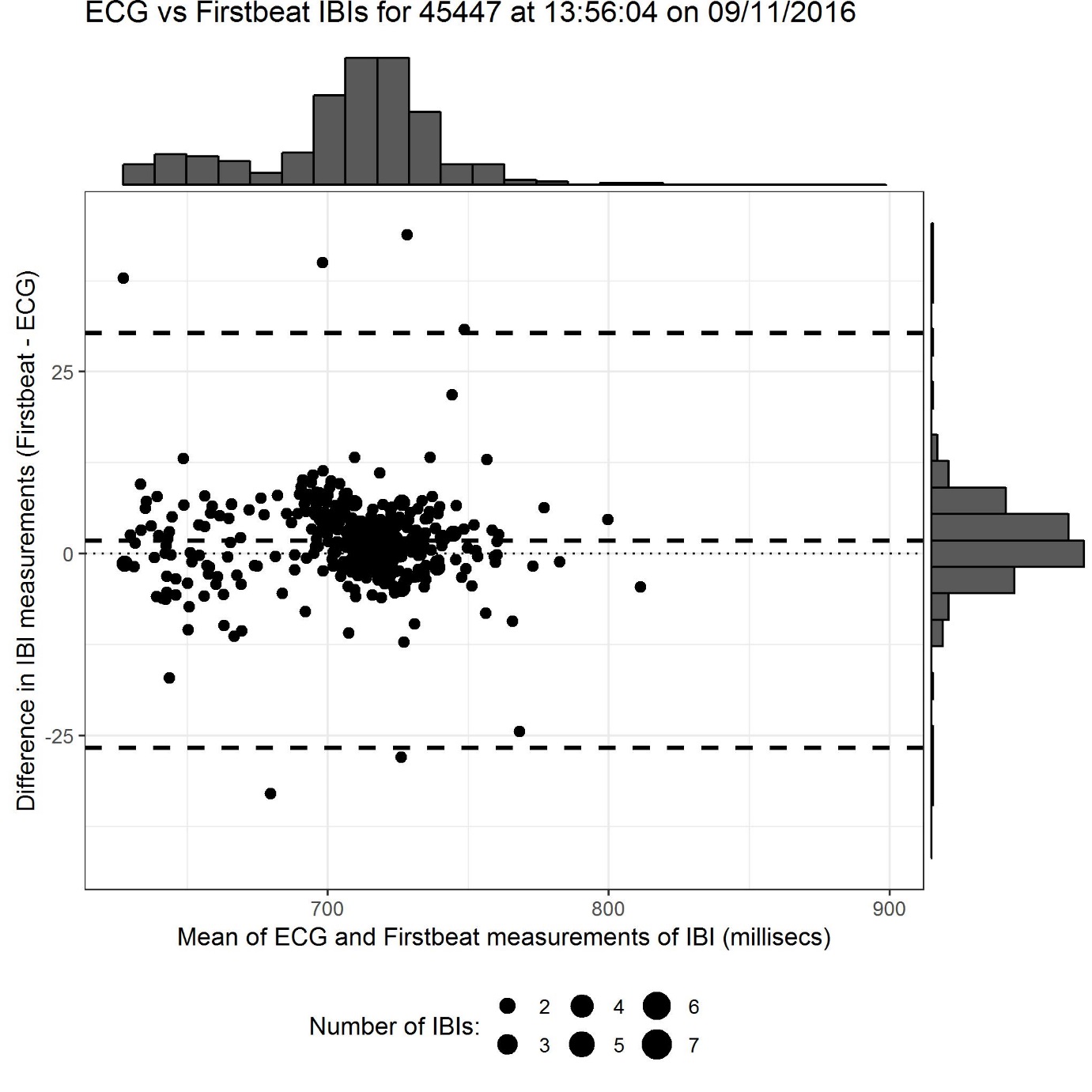


**S7 Fig. Bland-Altman plot showing limits of agreements between concurrent IBI data derived from the Firstbeat™ system and the AliveCor ECG for female ID3 at 13:56:04 GMT on 09/11/2016.**

**S1 Table. Summary statistics showing level of agreement within the 16 cases of concurrent ECG and Firstbeat™ IBI data.** Additional summary statistics from the correlational and Bland-Altman analyses used to validate the Firstbeat™ IBI data against in field ECG recordings. S2 Fig, S3 Fig, S6 Fig and S7 Fig illustrate examples of the high degree of agreement between IBI values derived from the in-field recordings by the Firstbeat™ system and the ECG recordings.

| **Parameter** | **mean** | **se** | **min** | **max** |
| --- | --- | --- | --- | --- |
| R (Pearson's) (for ECG vs Firstbeat™ correlation) | 0.89 | 0.03 | 0.46 | 1.00 |
| P (Pearson's) (for ECG vs Firstbeat™ correlation) | < 0.0001 | < 0.0001 | < 0.0001 | < 0.0001 |
| mean RR interval in each of 16 ECG traces | 635.43 | 12.57 | 556.34 | 717.86 |
| standard error for RR intervals in each of 16 ECG traces | 1.77 | 0.38 | 0.46 | 6.52 |
| mean difference (ms) between ECG and Firstbeat™ measures | 0.7 | 0.27 | -0.53 | 2.92 |
| standard error of the differences (ms) between ECG and Firstbeat™ measures | 0.51 | 0.07 | 0.2 | 1.34 |
| number of RR intervals | 155.44 | 29.05 | 24 | 422 |
| Lower limits of agreement | -10.17 | 1.42 | -27.27 | -4.16 |
| Upper limits of agreement | 11.58 | 1.54 | 5.72 | 30.9 |
| Lower 95% CI of the mean difference (bias) | -0.15 | 0.29 | -1.6 | 2.39 |
| Upper 95% CI of the mean difference (bias) | 1.55 | 0.3 | 0.06 | 3.45 |
| Lower 95% CI of the Lower limit of agreement | -11.65 | 1.51 | -29.3 | -5.15 |
| Upper 95% CI of the Lower limit of agreement | -8.7 | 1.36 | -25.25 | -2.39 |
| Lower 95% CI of the Upper limit of agreement | 10.1 | 1.48 | 5.13 | 28.88 |
| Upper 95% CI of the Upper limit of agreement | 13.05 | 1.63 | 6.3 | 32.92 |

**S2 Table. Retained GAMMs within the confidence sets for predicting (i) number of flats (log transformed), (ii) stairs (log transformed) and (iii) artefacts (square root transformed) in 5 min heart rate traces.** Model details: n(traces) = 6609, n(individuals) = 29, n(Heart rate monitor ID) = 17. s = smoothed terms. ID, Heart rate monitor ID and year included as random smooth terms.

| **Response variable** | **Model structure** | **df** | **AIC** | **ΔAIC** | **Weight** |
| --- | --- | --- | --- | --- | --- |
| **No. of flats** | s(Activity) + s(Artefacts%) + Time + Temp + s(Day) + s(tDeploy) +ID + Heart rate monitor ID | 62 | 17239 | 0 | 0.291 |
|  | s(Activity) + s(Artefacts%) + Time + s(Day) + s(tDeploy) + ID + Heart rate monitor ID + year | 62 | 17240.3 | 1.22 | 0.158 |
|  | s(Activity) + s(Artefacts%) + Time + s(Day) + s(tDeploy) + ID + Heart rate monitor ID | 61 | 17243.5 | 4.50 | 0.031 |
|  |  |  |  |  |  |
| **No. of stairs** | s(Activity) + s(Artefacts%) + s(Temp) + ID | 41 | 16987.7 | 0 | 0.108 |
|  | s(Activity) + s(Artefacts%) + s(Day) + s(Mass) + ID | 45 | 16988.8 | 1.09 | 0.063 |
|  |  |  |  |  |  |
| **No. of artefacts** | s(Activity) + s(Mass) + Time + Temp + s(Day) + s(tDeploy) +ID + Heart rate monitor ID | 65 | 31881.5 | 0 | 0.324 |
|  | s(Activity) + Time + Temp + s(Day) + s(tDeploy) +ID + Heart rate monitor ID | 62 | 31883.3 | 1.76 | 0.134 |
|  | s(Activity) + s(Mass) + Time + s(Day) + s(tDeploy) +ID + Heart rate monitor ID | 60 | 31886.1 | 4.61 | 0.032 |

**Notes:** Activity is the percentage of IBIs within each trace where seal was classified as ‘active. Artefacts is the percentage of IBI’s that were corrected for artefacts by the Firstbeat™ software in each trace. Temperature (Temp) in ^o^C measured at the nearest 30 min to the time of the 5 min heart rate trace onset. Time expressed as a proportion of 24 hr. Day expressed as number of days from 1^st^ January. tDeploy expressed as number of days since attachment of heart rate monitor to the individual seal (day 1 = day of attachment). Mass is the seal’s estimated mass (kg) on the day that the trace was recorded.

END
